# An efficient combination of BEST and NUS methods in multidimensional NMR spectroscopy for high throughput analysis of proteins†

**DOI:** 10.1039/c8ra00527c

**Published:** 2018-05-15

**Authors:** Veera Mohana Rao Kakita, Mandar Bopardikar, Vaibhav Kumar Shukla, Kavitha Rachineni, Priyatosh Ranjan, Jai Shankar Singh, Ramakrishna V. Hosur

**Affiliations:** UM-DAE Centre for Excellence in Basic Sciences, University of Mumbai Kalina Campus, Santacruz Mumbai 400 098 India; Department of Chemical Sciences, Tata Institute of Fundamental Research (TIFR) 1-Homi Bhabha Road, Colaba Mumbai 400 005 India hosur@tifr.res.in; Department of Biosciences & Bioengineering, Indian Institute of Technology-Bombay (IIT-B) Mumbai 400076 India

## Abstract

Application of Non Uniform Sampling (NUS) along with Band-selective Excitation Short-Transient (BEST) NMR experiments has been demonstrated for obtaining the important residue-specific atomic level backbone chemical shift values in short durations of time. This application has been demonstrated with both well-folded (ubiquitin) and unfolded (α-synuclein) proteins alike. With this strategy, the experiments required for determining backbone chemical shifts can be performed very rapidly, *i.e.*, in ∼2 hours of spectrometer time, and this data can be used to calculate the backbone folds of proteins using well established algorithms. This will be of great value for structural proteomic investigations on one hand, where the speed of structure determination is a limiting factor and for application in the study of slow kinetic processes involving proteins, such as fibrillization, on the other hand.

## Abstract 2

Application of NUS along with BEST NMR experiments has been demonstrated for obtaining the important residue-specific atomic level backbone chemical shift values in short durations of time.

## Graphical abstract

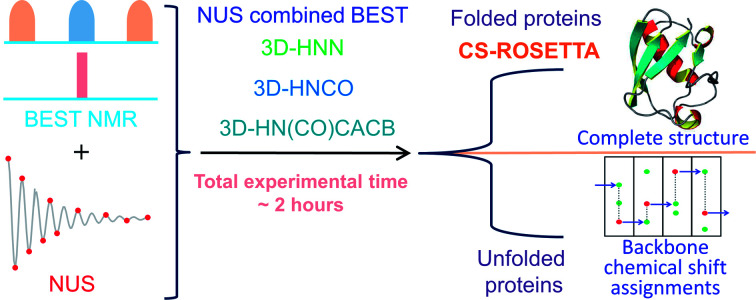

## 1. Introduction

Solution-state NMR spectroscopy has made great progress over the past three decades in enabling determination of structural and dynamic properties of non-crystalline and soluble proteins.^1^ In this endeavour, chemical shift assignment is a prerequisite as it provides valuable atomic level information, which can be used for understanding not only the structural^2–5^ and dynamical properties^3–7^ of proteins, but also a vast variety of protein–protein interactions,^8^ which are crucial for biological functions. Conventionally, the process involves recording a set of nD-experiments for deriving backbone and side-chain chemical shift assignments followed by analysis of NOESY-derived distances or subjecting the chemical shift assignments to online servers for structural elucidation; the latter strategy of deriving structural information using chemical shift alone is rather fast as compared to the former one, although it only provides backbone folds. Side-chain chemical shift information is extremely useful for understanding protein–ligand and protein–protein interactions.^9^

One of the difficulties commonly encountered in the above-mentioned experiments is that under physiological conditions, stability of some proteins is rather poor due to many reasons such as self-proteolysis,^10^ thermal instability,^11^ oligomerization,^12^ and aggregation.^13^ In such situations, the required multi-dimensional NMR measurements have to be recorded within the life span of the native state of the protein under study. In this context, several strategies have been proposed: Filter Diagonalisation Method (FDM),^14^ Reduced Dimensionality (GFT^15,16^ and APSY^17^), Non-Uniform Sampling (NUS),^18–22^ projection reconstruction,^23^ Shaped Arrayed data acquisition protocol (SHARC NMR),^24^ Hadamard NMR,^25^ Ultrafast,^26^ Covariance NMR,^27^ multiple-receiver techniques,^28–32^ longitudinal relaxation optimization,^33,34^ cooling overall spin temperature (COST),^35^ HSQC-based multi-dimensional out-and-back experiments,^36^ Band-Selective Optimized-Flip-Angle-Short-Transient (SOFAST),^37^ and Band-selective Excitation Short-Transient (BEST)^38^ methods, which help to reduce the nD-NMR experimental times to a significant extent. Atreya *et al.*^34^ have demonstrated the importance of combined implementation of two fast NMR techniques (longitudinal relaxation optimization^33^ and GFT^15^) for obtaining the chemical shift information in short instrumental times. However, to analyse the chemical shifts from the GFT-acquired spectra, sub-spectra need to be generated by performing appropriate linear combinations.

Among the various options discussed above, BEST and NUS have been the more commonly used techniques. BEST uses band-selective pulses and thus exhibits very small recycle delays. NUS requires special sampling schedules as well as processing schemes,^18–22^ which are readily available on most spectrometers. Thus, a combination of these two fast data acquisition methods can turn out to be a method of choice, which may open up new avenues for time-resolved atomic resolution studies of kinetic processes involving proteins. Till date, there have been very few articles reporting such studies, which either utilize a set of hyper-dimensional NMR experiments or require acquisition of many three-dimensional NMR experiments.^39–43^ Although, in general, BEST and NUS strategies can be incorporated in every pulse sequence, one would like to specifically record a minimal set of experiments, namely, BEST- (HNCO, HN(CA)CO, HNCA, HN(CO)CA, HNCACB, and HN(CO)CACB) in combination with NUS, so that the obtained chemical shift values can be fed into either CS-ROSETTA^44^ or CS23D^45^ server to obtain the three dimensional structure of the protein of interest. Interestingly, a similar approach has been suggested for the determination of atomic resolution structures of the so-called excited states of proteins.^46^

Another difficulty one often faces while dealing with the biologically important intrinsically disordered/partially folded proteins is the poor ^1^HN chemical shift dispersion in these systems. In such cases, 3D-HNN type of experiments (HNN,^47^ A-HNN,^48^ and ST-HNN^49^) have proved to be very useful as they provide correlations between well-resolved ^15^N sites of adjacent residues (N_*i*−1_–N_*i*_–N_*i*+1_) and additionally, these spectra display different signals for the diagonal (N_*i*_) and cross-peaks (N_*i*+1_ and N_*i*−1_). Besides, they also display triplet specific peak patterns, which serve as checkpoints during the peak assignment process. In fact, HNN has also proved to be quite useful even for small folded proteins. However, one of the shortcomings of these experiments is that they are less sensitive and thus require high data acquisition times for signal averaging.

The present manuscript offers a solution to the above-discussed problems in the form of a set of NUS-combined BEST-HNN,^50^ BEST-HNCO and BEST-HN(CO)CACB^38^ experiments for the backbone chemical shift assignments: –^1^HN, –^15^N, –CO, –Cα and –Cβ of folded as well as unfolded proteins. We can consider this set of methods as protein friendly NMR experiments, as they help in high throughput analysis of both folded and unfolded proteins in a rather short experimental time of about 2 h for recording all the experiments. The utility of NUS-combined BEST experiments has been demonstrated for both intrinsically disordered α-synuclein as well as well-folded ubiquitin proteins.

α-Synuclein (α-syn) is a 140 amino acid residue long intrinsically disordered protein (IDP). This protein accumulates in the cytoplasm of dopaminergic neurons, leading to the formation of lewy bodies (LB) (containing fibrillar α-syn), which finally causes neurodegenerative disorders such as Parkinson's disease (PD), lewy body dementia (LBD), and multiple system atrophy (MSA).^51,52^ Familial early onset PD is associated with the duplication or triplication of the gene encoding α-syn (SNCA).^53^ Various point mutations in SNCA relate to autosomal dominant familial form of PD.^54–56^ The oligomers of α-syn are thought to be more toxic to dopaminergic neurons than α-syn fibrils probably due to their ability to puncture the cell membrane, which causes disruption in Ca^2+^ homeostasis.^57^ These oligomers are on-pathway intermediates, which occur during the fibrillization of α-syn. The overall process of fibrillization of α-syn takes place over a period of few days; however, the time-scale of conformational changes leading up to the toxic oligomers is only a few hours.^58^ Therefore, to obtain an atomic level understanding of α-syn conformation present, it is necessary to record 3D NMR spectra within the lifespan of the conformational species, *i.e.*, a few hours so that backbone chemical shift assignments are determined; now, this represents a significant challenge. In this study, we have addressed this challenge by performing the 3D BEST-HNN experiment on α-syn with the help of non-uniform sampling within ∼1.5 h.

Ubiquitin, a 76 amino acid residue long globular or folded protein is the earliest known member of a structurally conserved family of proteins that are known to regulate a wide variety of processes in eukaryotic cells. This protein has been the subject for extensive studies in the recent years^59^ due to its involvement in diverse biochemical processes.

## 2. Materials and methods

### 2.1 Expression and purification of proteins

Both α-syn and ubiquitin proteins were overexpressed in BL21 (DE3) *E. coli* cells, as described elsewhere,^60^ with the modification that the cells were grown in M9 minimal media containing ^15^N-ammonium chloride and ^13^C-glucose as the only source of nitrogen and carbon, respectively, for the generation of uniformly labelled ^13^C/^15^N samples. α-Syn was purified according to the guidelines illustrated in detail by Volles *et al.*^60^ His-tagged ubiquitin was affinity purified on Ni-NTA beads (Sigma-Aldrich) and eluted with Tris buffer (pH 7.5) containing 250 mM imidazole.

### 2.2 NMR measurements

All the experiments on α-syn were performed at 288 K on a Bruker Avance 600 MHz NMR spectrometer with a cryogenically cooled probe. The experiments on ubiquitin were carried out at 298 K on a Bruker Avance 750 MHz NMR spectrometer equipped with a room temperature probe. ^13^C/^15^N-labelled α-syn protein sample was prepared at a concentration of ∼800 μM at pH 6.0 in 20 mM phosphate buffer containing 90% H_2_O and 10% D_2_O. ^13^C/^15^N-labelled ubiquitin sample was prepared at a concentration of ∼1 mM at pH 5.0 in 25 mM acetate buffer containing 90% H_2_O and 10% D_2_O (see ESI† for further experimental details).

## 3. Results and discussion

We have previously demonstrated the advantages of BEST-HNN over HNN with regard to the S/N ratio under conditions of identical resolutions and experimental times.^50^ Herein, we first try to define the appropriate number of increments for achieving acceptable resolution to enable the assignments, keeping in mind the requirement of short experimental time. Following this, we have compared the performances of non-uniform sampling (NUS) and uniform sampling (US)-based BEST-HNN experiments.

**Fig. 1:**
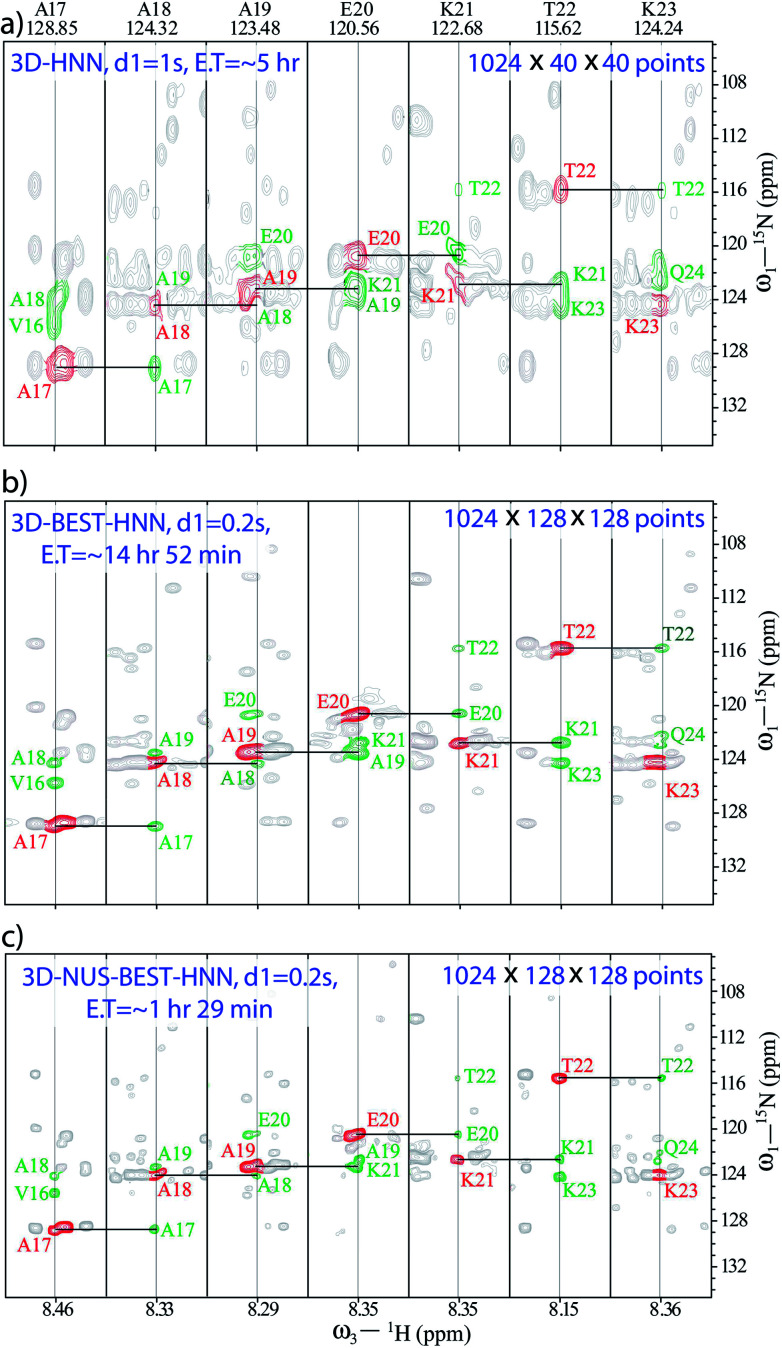
Comparison of 3D-HNN spectra of α-synuclein recorded in different experimental schemes; (a) conventional-HNN, (b) BEST-HNN, and (c) NUS-BEST-HNN. Herein, unambiguous 15N chemical shift assignments have been achieved with the aid of 3D-NUS-BEST-HNN.

Fig. 1 compares the 3D-HNN spectra of α-syn recorded with different versions of pulse schemes and with different parameters. The conventional HNN experiment was recorded in (8 scans and 1024 × 40 × 40 number of points, with 1 s of recycle delay) ∼5 h of experimental time (Fig. 1a). Herein, ^15^N chemical shift resolution was quite poor due to the acquisition of less number of dwell increments. As a result, establishing ^15^N chemical shift correlation was almost impossible for the residues A19-K21, wherein closely separated cross-peak intensities with negative signals were cancelled out by the positive diagonal peaks. In fact, to resolve such peaks for having good ^15^N chemical shift resolution, 3D-HNN experiments required more number of points in the indirect dimensions; for example, in the present case, the experiment needed about 128 increments in both the indirect dimensions. As a result, 3D-HNN experiment had to be acquired for ∼2 days, which was much longer than the lifetime of the native state of an IDP under aggregating conditions.^58^

In such cases, recording BEST version of HNN experiment is a good choice as it quickly provides the direct ^15^N chemical shift correlations, and a single experiment is adequate to get the whole set of amino acids present. Thus, a BEST-HNN experiment was acquired in ∼14 h of spectrometer time, with 0.2 s of recycle delay and 128 dwelling increments in both the indirect dimensions (Fig. 1b). In the BEST-HNN spectrum, very closely separated ^15^N chemical shifts were satisfactorily resolved, and this allowed unambiguous identification of A19-K21 residues. However, ∼14 h of experimental time was still too long.

Therefore, to reduce data acquisition time, we implemented NUS (10% random sampling); here, selection of random sampling was due to the constant-time evolution along both the indirect dimensions of HNN. This reduced the experimental time to only ∼1 h 29 min, which was a substantial improvement over the experimental times of other HNN pulse schemes. The BEST-HNN experiments were acquired with 10%, and 20% NUS sampling, and we found that 10% sampling was adequate for reproducing exact results. Here, the NUS-BEST-HNN data sets were processed by using various protocols, *viz.*, MDD (multi-dimensional decomposition),^61^ IST (iterative soft threshold),^62^ and IRLS (iteratively re-weighted least squares)^62^ in Bruker Topspin 4.0.1. Finally, chemical shift analysis was performed for the IRLS-processed NUS-BEST-HNN data set in the CARA software,^63^ as the spectral quality of IRLS-processed data set was found to be superior (with negligible artefacts) compared to those of the other two processing protocols (see ESI Fig. 1 and 2†). Subsequently, signal-to-noise ratios (SNRs) for all the amide functional groups were also measured, and a good agreement was noticed between the BEST-NUS and NUS-BEST-HNN spectra (see ESI Fig. 3†). Similarly, analysis of D98-N103 residues was also performed with the help of the NUS-BEST-HNN experiment (see ESI Fig. 4†). The acquisition time of the NUS-BEST-HNN experiment (∼1 h 29 min) was well within the lifetime of the native state of aggregating α-syn, and this makes it an incredible tool for atomic resolution structural investigations on aggregating IDPs in general.

**Fig. 2:**
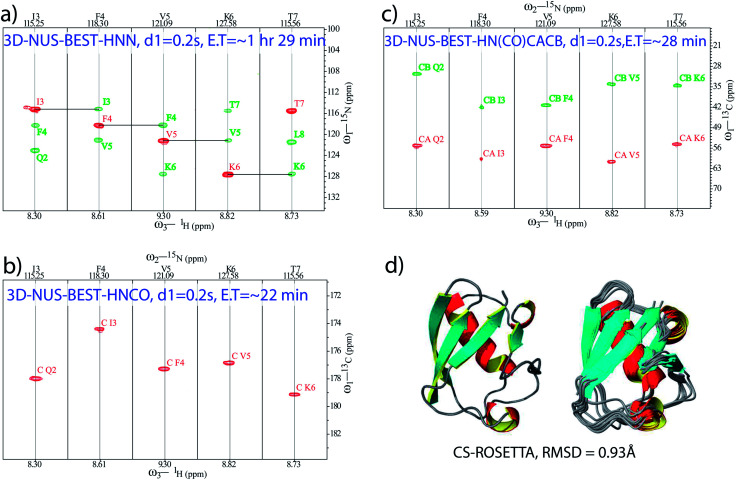
NUS-BEST-HNN, NUS-BEST-HNCO, and NUS-BEST-HN(CO)CACB spectra recorded for ubiquitin protein are shown in (a), (b) and (c), respectively. Herein, assigned backbone chemical shift values were subjected to the CS-ROSETTA calculations, and the obtained three dimensional structure with satisfactory convergence (RMSD = 0.93 Å for the backbone) is depicted in (d).

Apart from fast backbone H^N^ and N chemical shift assignments for an aggregating IDP, attempts were made for the development of a protocol for rapid structural elucidation of small well-folded proteins. As an example, a set of NUS-combined BEST schemes (with 0.2 s of relaxation delay), namely, BEST-HNN (10% sampling, Fig. 2a), BEST-HNCO (5% sampling, Fig. 2b), and BEST-HN(CO)CACB (5% sampling, Fig. 2c) experiments were recorded on a BRUKER 600 MHz spectrometer in ∼1 h 29 min, ∼22 min, and ∼28 min, respectively, on a ^13^C and ^15^N doubly labelled ubiquitin protein (see ESI† for the further details). Indeed, 5% NUS sampling in both these BEST-HNCO and BEST-HN(CO)CACB experiments was found to be sufficient to observe all the peaks. In this case, the set of NUS-BEST experiments together required only ∼2 h 20 min of spectrometer time.

Subsequently, the backbone ^1^H, ^15^N, CO, Cα, and Cβ chemical shifts (total of 344) obtained from these experiments (from the NUS-BEST experiments) were subjected to the CS-ROSETTA structure calculations,^44^ which generated a structure with RMSD values of 0.93 Å and 1.3 Å for the backbone and heavy atoms, respectively, (Fig. 2d). The present protein structure had an RMSD of 0.8 Å with respect to the NMR structure reported earlier (PDB ID: 1D3Z) while using only ordered residues for the calculations (see ESI Fig. 5†). On the other hand, relative to the same reference structure (1D3Z), the present CS-ROSETTA structure showed backbone and heavy atom RMSD values of 1.0 Å, and 1.4 Å, respectively. These values were found to be in good agreement with the backbone (0.75 Å) and heavy atom (1.35 Å) RMSD values reported by Shen *et al.* relative to the NMR structure 1D3Z.^44^ These RMSD values were calculated from the PSVS server (http://www.psvs-1_5-dev.nesg.org/) and the program PYMOL (http://www.pymol.sourceforge.net/).

In conclusion, the present study demonstrates a protocol employing combined application of BEST- and NUS-based fast acquisition techniques for quick investigations into the structural features of small-sized well-folded proteins as well as intrinsically disordered proteins. We have recorded a minimal set of BEST-HNN, BEST-HNCO and BEST-HN(CO)CACB experiments in only ∼2 h 20 min of spectrometer time for ubiquitin protein. The obtained backbone chemical shift values when subjected to the CS-ROSETTA analysis provide a three-dimensional structure of the protein, which is found to closely resemble the previously reported structure obtained by the conventional lengthy procedures based on NOE structural restraints. The protocol proposed here makes fairly accurate structural elucidation of small folded proteins possible in a day's time, which is a great advancement over the conventional methods. Also, the method makes it feasible to determine structural information for proteins at lower concentrations than those required by the contemporary methods, because the gain in the sampling rate due to NUS can be used to acquire more number of scans. As an important application, the quick determination of atomic level (backbone ^15^N and ^1^H chemical shifts) information of intrinsically disordered α-syn in only ∼1 h 29 min from a single experiment, NUS-BEST-HNN, in combination with prior knowledge of the amino acid sequence of the protein presents new prospects for identification of factors contributing majorly to the reaction coordinates of the aggregation process. Taking into consideration the pace at which backbone chemical shift information is made available by this approach, our results seem to present exciting opportunities for tracking the aggregation pathways of IDPs at atomic resolution in a time-dependent manner, which may provide deeper insights into these pathophysiological pathways undertaken by IDPs, and this can consequently lead to the development of novel therapeutics for neurodegenerative disorders; this will be the subject matter of a separate publication in the near future.

## Conflicts of interest

The authors declare no conflict of interest.

## Supplementary Material

RA-008-C8RA00527C-s001
