# Supplementary material for: An efficient combination of BEST and NUS methods in multidimensional NMR spectroscopy for high throughput analysis of proteins
Source: RSC Adv. 2018 May 15;8(32):17616–21. doi: 10.1039/c8ra00527c (PMC9080477; doi:10.1039/c8ra00527c)
Supplement: RA-008-C8RA00527C-s001 [file RA-008-C8RA00527C-s001.pdf]

Electronic Supplementary Material (ESI) for RSC Advances.
This journal is © The Royal Society of Chemistry 2018

Supporting Information

An Efficient Combination of BEST and NUS Methods in Multidimensional NMR Spectroscopy: High Throughput Analysis of Proteins

Experimental details of α-Synuclein protein (600 MHz)

Experiment: 3D-HNN
Sample: α-Synuclein
Number of Scans: 8
Recycle Delay: 1s
Experimental Time: ~5 hr 20 min

Dimension	Acquisition parameters						Processing parameters
	Size of FID	Spectral Width (Hz)	Acquisition time (sec)	FID resolution (Hz)	Spectrometer Frequency (MHz)	Transmitter Frequency Offset (Hz)	Number of points filled
F3	1024	7211.54	0.071	14.1	600.4	4.7	1024
F2	40	2129.59	0.0094	106.5	60.84	119.0	256
F1	40	2129.59	0.0094	106.5	60.84	119.0	256

Experiment: 3D-BEST-HNN
Sample: α-Synuclein
Number of Scans: 8
Recycle Delay: 0.2s
Experimental Time: ~14 hr 52 min

Dimension	Acquisition parameters						Processing parameters
	Size of FID	Spectral Width (Hz)	Acquisition time (sec)	FID resolution (Hz)	Spectrometer Frequency (MHz)	Transmitter Frequency Offset (Hz)	Number of points filled
F3	1024	7211.54	0.071	14.1	600.4	4.7	1024
F2	128	2129.59	0.03	33.3	60.84	119.0	256
F1	128	2129.59	0.03	33.3	60.84	119.0	256

Experiment: 3D-NUS-BEST-HNN (10% Random sampling)
Sample: α-Synuclein
Number of Scans: 8
Recycle Delay: 0.2s
Experimental Time: ~1 hr 29 min

Dimension	Acquisition parameters						Processing parameters
	Size of FID	Spectral Width (Hz)	Acquisition time (sec)	FID resolution (Hz)	Spectrometer Frequency (MHz)	Transmitter Frequency Offset (Hz)	Number of points filled
F3	1024	7211.54	0.071	14.1	600.4	4.7	1024
F2	128	2129.59	0.03	33.3	60.84	119.0	256
F1	128	2129.59	0.03	33.3	60.84	119.0	256

Experimental details of Ubiquitin protein (600 MHz / 750 MHz)

Experiment: 3D-BEST-HNN
Sample: Ubiquitin
Number of Scans: 8
Recycle Delay: 0.2s
Experimental Time: ~14 hr 52 min

Dimension	Acquisition parameters						Processing parameters
	Size of FID	Spectral Width (Hz)	Acquisition time (sec)	FID resolution (Hz)	Spectrometer Frequency (MHz)	Transmitter Frequency Offset (Hz)	Number of points filled
F3	1024	7211.54	0.071	14.1	600.4	4.7	1024
F2	128	2129.59	0.03	33.3	60.84	116.5	256
F1	128	2129.59	0.03	33.3	60.84	116.5	256

Experiment: 3D-BEST-HNCO
Sample: Ubiquitin
Number of Scans: 8
Recycle Delay: 0.2s
Experimental Time: ~7 hr 23 min

Dimension	Acquisition parameters						Processing parameters
	Size of FID	Spectral Width (Hz)	Acquisition time (sec)	FID resolution (Hz)	Spectrometer Frequency (MHz)	Transmitter Frequency Offset (Hz)	Number of points filled
F3	698	10504.2	0.033	30.1	750.28	4.7	1024
F2	96	2737.23	0.0175	57.0	76.03	116.5	256
F1	96	2452.98	0.0196	51.1	188.66	172.5	256

Experiment: 3D-BEST-HN(CO)CACB
Sample: Ubiquitin
Number of Scans: 8
Recycle Delay: 0.2s
Experimental Time: ~7 hr 58 min

Dimension	Acquisition parameters						Processing parameters
	Size of FID	Spectral Width (Hz)	Acquisition time (sec)	FID resolution (Hz)	Spectrometer Frequency (MHz)	Transmitter Frequency Offset (Hz)	Number of points filled
F3	698	10504.2	0.033	30.1	750.28	4.7	1024
F2	96	2737.23	0.0175	57.0	76.03	116.5	256
F1	96	2452.98	0.0196	51.1	188.66	41.0	256

Experiment: 3D-NUS-BEST-HNN (10% Random sampling)<br>Sample: Ubiquitin<br>Number of Scans: 8<br>Recycle Delay: 0.2s<br>Experimental Time: ~1 hr 29 min							
Dimension	Acquisition parameters						Processing parameters
	Size of FID	Spectral Width (Hz)	Acquisition time (sec)	FID resolution (Hz)	Spectrometer Frequency (MHz)	Transmitter Frequency Offset (Hz)	Number of points filled
F3	1024	7211.54	0.071	14.1	600.4	4.7	1024
F2	128	2129.59	0.03	33.3	60.84	116.5	256
F1	128	2129.59	0.03	33.3	60.84	116.5	256

Experiment: 3D-NUS-BEST-HNCO (5% Random sampling)<br>Sample: Ubiquitin<br>Number of Scans: 8<br>Recycle Delay: 0.2s<br>Experimental Time: ~22 min							
Dimension	Acquisition parameters						Processing parameters
	Size of FID	Spectral Width (Hz)	Acquisition time (sec)	FID resolution (Hz)	Spectrometer Frequency (MHz)	Transmitter Frequency Offset (Hz)	Number of points filled
F3	698	10504.2	0.033	30.1	750.28	4.7	1024
F2	96	2737.23	0.0175	57.0	76.03	116.5	256
F1	96	2452.98	0.0196	51.1	188.66	172.5	256

Experiment: 3D-NUS-BEST-HN(CO)CACB (5% Random sampling)<br>Sample: Ubiquitin<br>Number of Scans: 8<br>Recycle Delay: 0.2s<br>Experimental Time: ~28 min							
Dimension	Acquisition parameters						Processing parameters
	Size of FID	Spectral Width (Hz)	Acquisition time (sec)	FID resolution (Hz)	Spectrometer Frequency (MHz)	Transmitter Frequency Offset (Hz)	Number of points filled
F3	698	10504.2	0.033	30.1	750.28	4.7	1024
F2	96	2737.23	0.0175	57.0	76.03	116.5	256
F1	96	2452.98	0.0196	51.1	188.66	41.0	256

Processing parameters: NUS-BEST-HNN spectra (on Topspin 4.0.1)

Processing method	Number of iterations	Processing time*
MDD	1000	7 hr
IST	10000	1 hr 50 min
IRLS	100	13 hr 40 min

* These are the typical processing times, when the data has been processed on a regular workstation connected with a Bruker NMR spectrometer. Indeed, they may reduce significantly, if we process the data on high power super computers/ cluster systems.

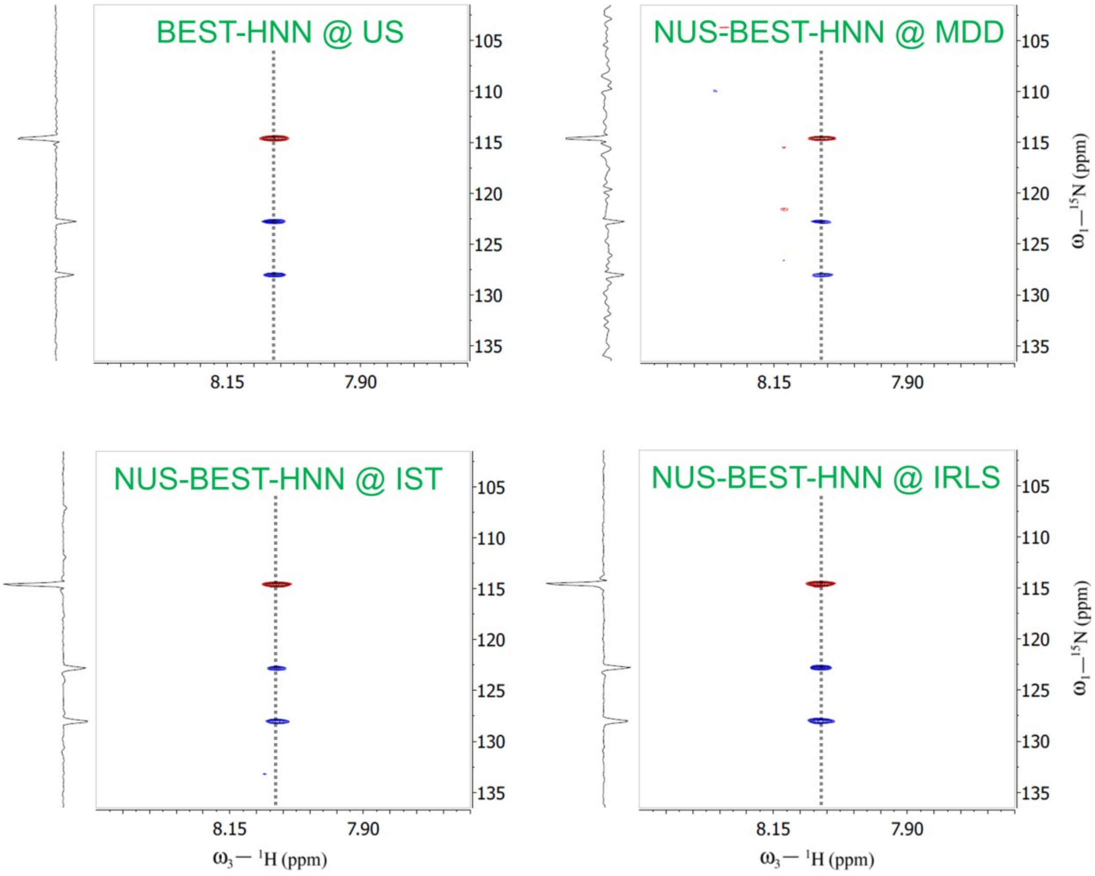

S. Figure. 1: Comparison of the 13-plane projections of BEST-HNN spectra recorded for α-Synuclein in uniformly sampled (US) and non-uniformly sampled (NUS) experimental conditions. Here, NUS-BEST-HNN has been processed in different processing schemes, viz, MDD, IST, and IRLS. The 1D-traces obtained at chemical shift of T54 residue have been shown in dotted lines and added as external projections. Further, these 1D-trances are compared in the S. Figure. 2.

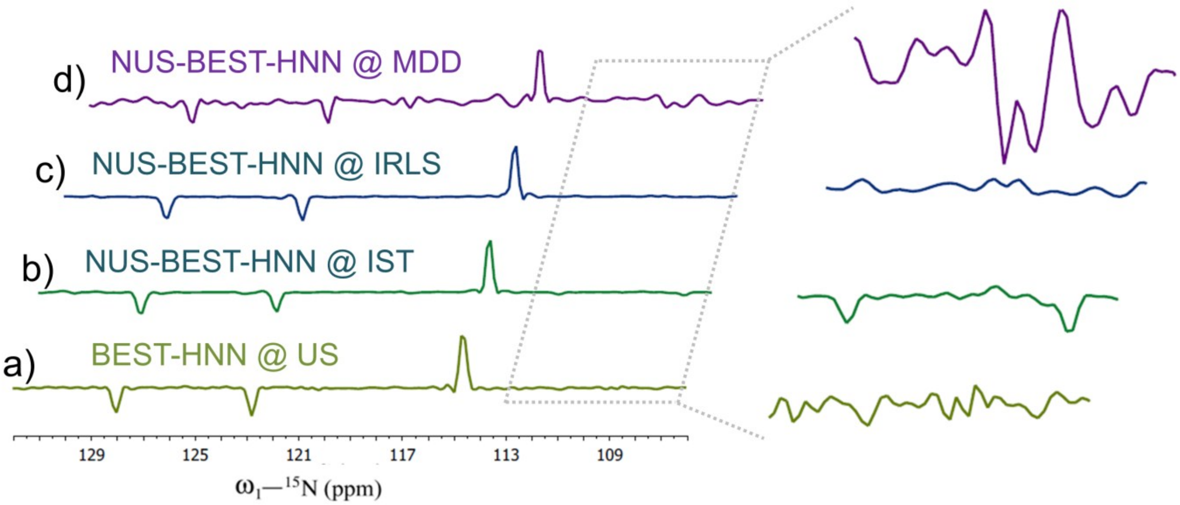

S. Figure. 2: Comparison of the 1D-traces of BEST-HNN spectra (T54 residue of 13-plane projection) recorded in uniformly sampled (US, a) and non-uniformly sampled (NUS, b, c, d) experimental conditions. Here, NUS-BEST-HNN has been processed in different processing schemes, viz, IST (b), IRLS (c), and MDD (d). A close comparison of the noise regions of these traces revealed that the IRLS processing has resulted in a very clean spectrum (free from false peaks), when compared with the other MDD and IST processing methods. Hence, we have adopted IRLS processing for all the NUS acquired spectra.

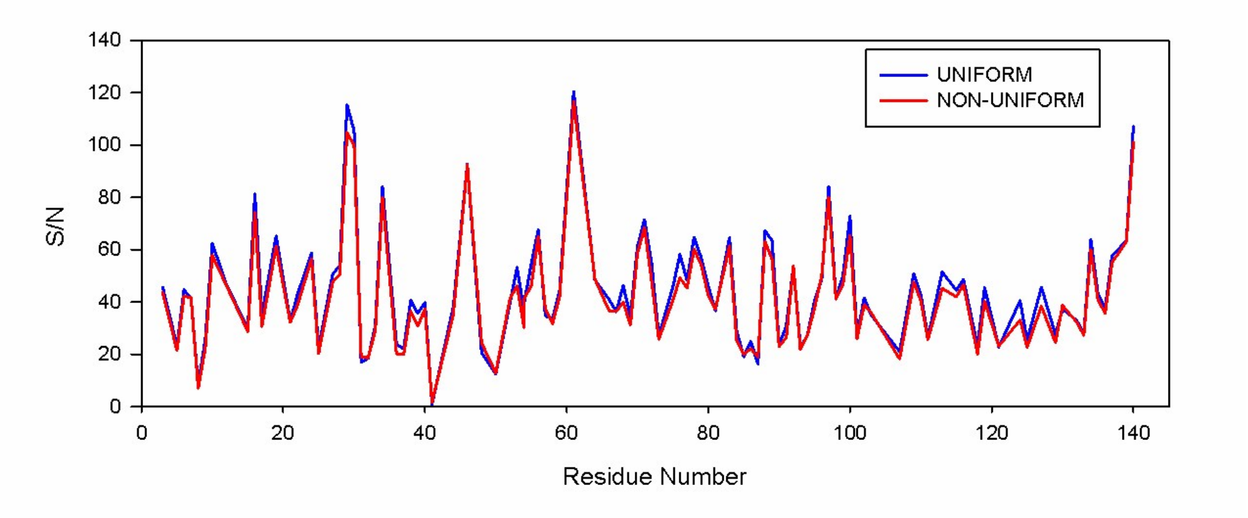

S. Figure. 3: Residue wise Signal to noise ratio (S/N) comparison (from BEST-HNN) for the α-Synuclein protein recorded in uniformly sampled and non-uniformly sampled acquisition modes. The diagonal peaks in the F1-F3 planes have been used; note that although the diagonals of Gly residues have negative intensities, the S/N ratio is represented as a positive number in every case.

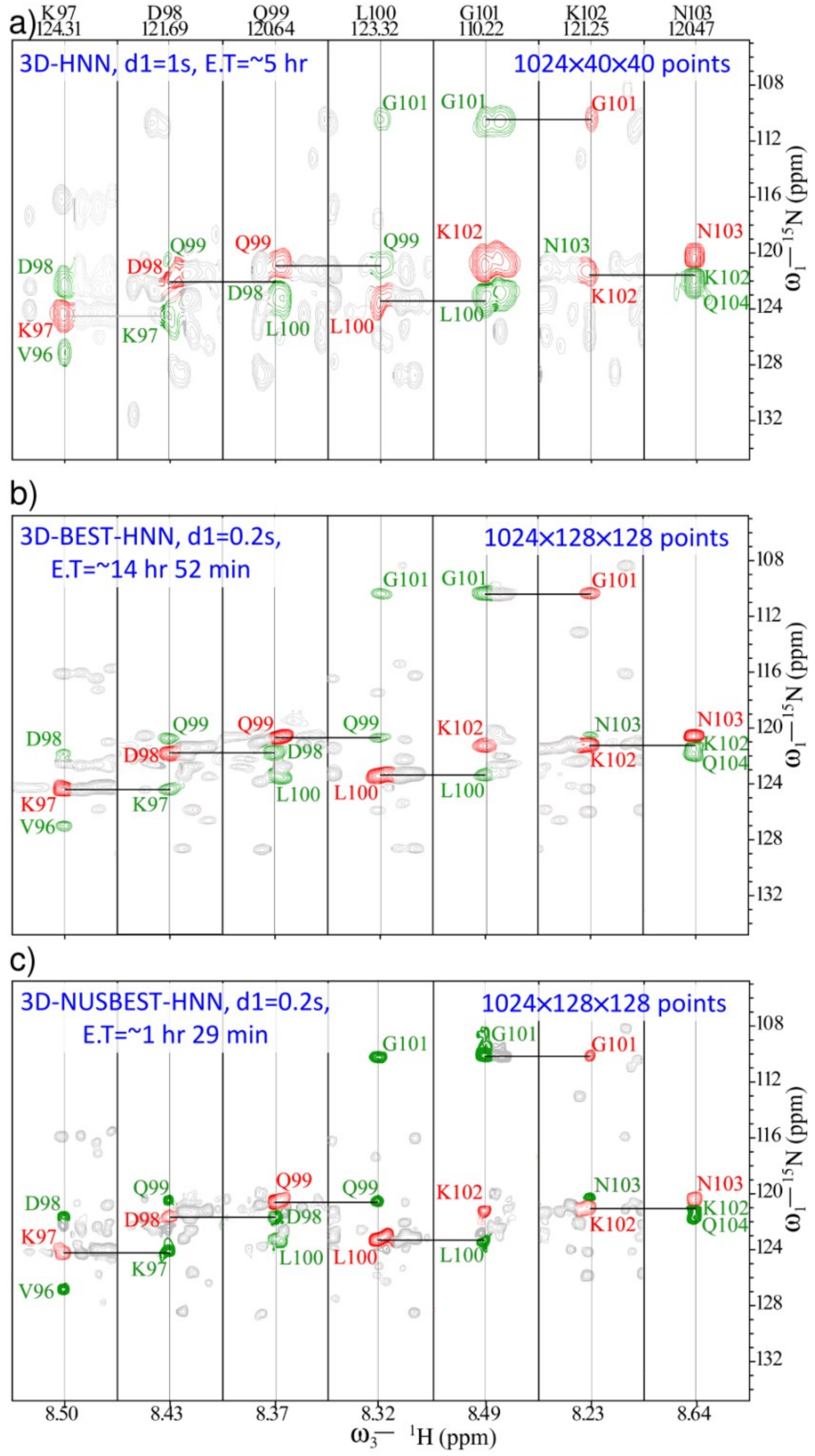

S. Figure. 4: Comparison of 3D-HNN spectra of α-Synuclein recorded in different experimental schemes; a) conventional-HNN, b) BEST-HNN, and c) NUS-BEST-HNN. Herein, unambiguous ^{15}N chemical shift assignments have been achieved with the aid of 3D-NUS-BEST-HNN, recorded in only ~ 1 hr 29 min of experimental time.

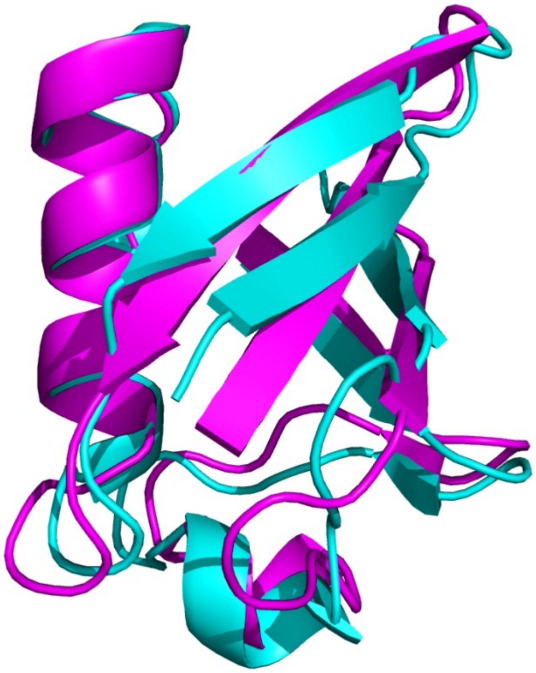

S. Figure. 5: Comparison of chemical shift based structure with the structure derived from NOE assignments. Chemical shift based (CS-ROSETTA) and NOE-derived structures of Ubiquitin (PDB ID: 1D3Z) are shown in magenta and cyan colors, respectively.

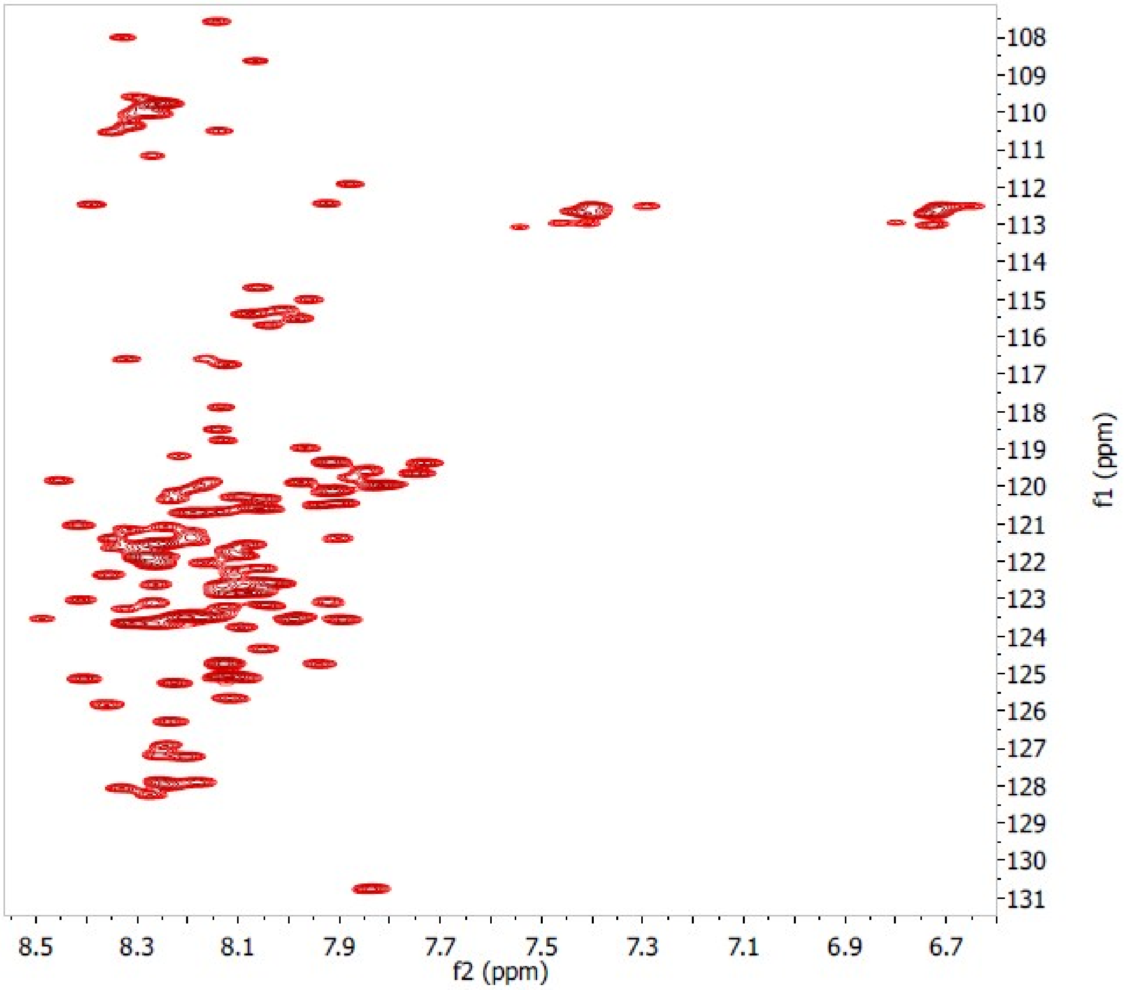

S. Figure.6: ^{1}H-^{15}N HSQC NMR spectrum of ^{15}N, ^{13}C labeled α-Synuclein.

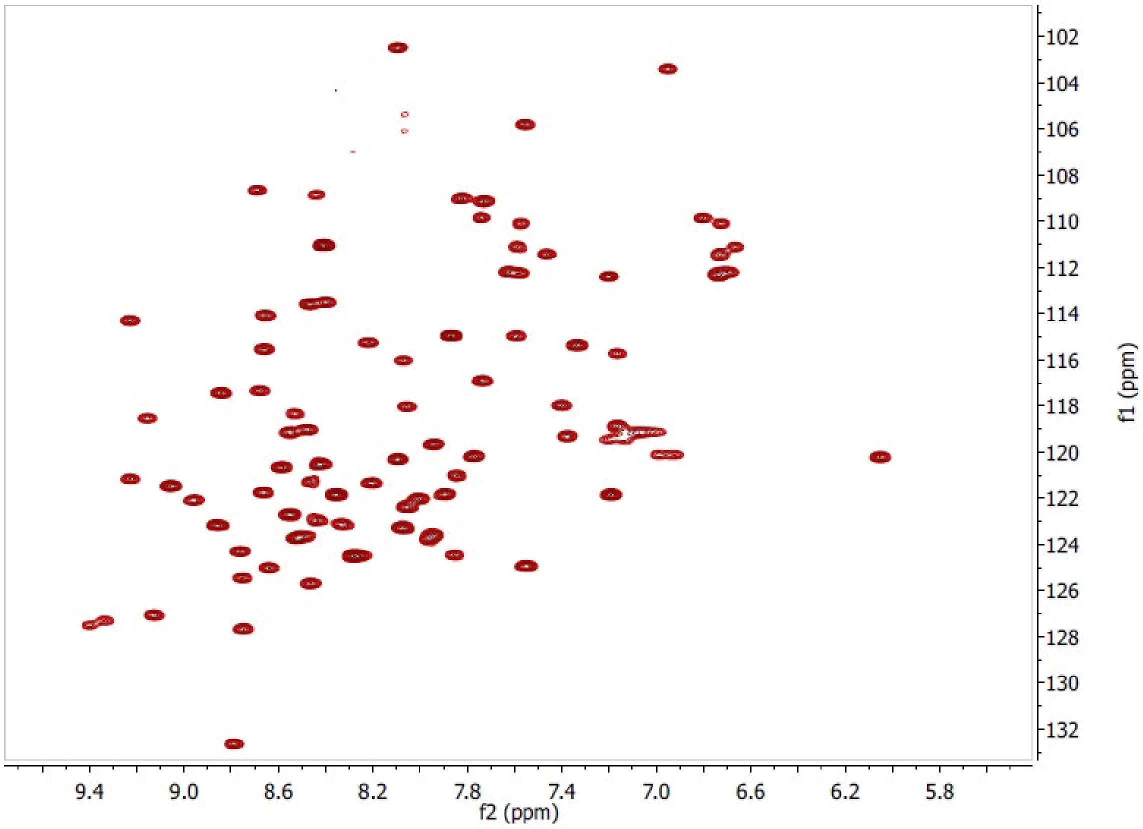

S. Figure.7: ^{1}H-^{15}N HSQC NMR spectrum of ^{15}N, ^{13}C labeled ubiquitin.
